# SP1 transcriptionally regulates UBE2N expression to promote lung adenocarcinoma progression

**DOI:** 10.1186/s43556-023-00118-2

**Published:** 2023-03-25

**Authors:** Jianjun Li, Chunchun Qi, Shanshan Shao, Yanru Chen, Zimei Peng, Qinglin Shen, Zhen Zhang

**Affiliations:** 1 Department of Pulmonary and Critical Care Medicine, The First Affiliated Hospital of Soochow University, Suzhou, 215006 China; 2 Suzhou Key Laboratory for Respiratory Diseases, Suzhou, 215006 China; 3 Institute of Respiratory Diseases, Soochow University, Suzhou, 215006 China; 4 Medical College of Nankai University, Tianjin, 300071 China; 5 Nanjing University of Chinese Medicine, Nanjing, 210023 China; 6 Institute of Clinical Medicine, Jiangxi Provincial People’s Hospital, The First Affiliated Hospital of Nanchang Medical College, 152 Aiguo Road, Nanchang, Jiangxi 330006 China; 7 Department of Oncology, Jiangxi Provincial People’s Hospital, The First Affiliated Hospital of Nanchang Medical College, Jiangxi Nanchang, 330006 China

**Keywords:** LUAD, UBE2N, SP1, Progression

## Abstract

Lung adenocarcinoma (LUAD) is the main cause of cancer-related death worldwide. Understanding the mechanisms of LUAD progression may provide insights into targeted therapy approaches for this malignancy. Ubiquitin-conjugating enzyme 2 N (UBE2N) has been demonstrated to play key roles in the progression of various cancers. However, the functions and mechanisms underlying UBE2N expression in LUAD are still unclear. In this study, we found that UBE2N is highly expressed in LUAD and patients with high UBE2N expression in their tumors have poor clinical outcomes. Moreover, we showed that UBE2N interference significantly inhibited LUAD progression in vitro and in vivo. At the molecular level, we demonstrated that the UBE2N is a bona fide target of transcription factor SP1. SP1 directly bound to the promoter of UBE2N and upregulated its expression in LUAD cells, which in turn contributed to the progression of LUAD. Furthermore, we found that there is a strong positive correlation between the expression of SP1 and UBE2N in LUAD samples. Importantly, LUAD patients with concomitantly high expression of SP1 and UBE2N were significantly associated with poor clinical outcomes. In conclusion, our study demonstrated that the SP1-UBE2N signaling axis might play a key role in the malignant progression of LUAD, which provides new targets and strategies for the treatment of LUAD.

**Supplementary Information:**

The online version contains supplementary material available at 10.1186/s43556-023-00118-2.

## Introduction

Lung cancer remains the most prevalent and deadliest cancer in the whole world, among which lung adenocarcinoma (LUAD) accounts for approximately 40% [1, 2]. Despite the improvement in treating LUAD, it is still the principal cause of cancer death in clinically diagnosed cases [3]. Thus, there is necessary to investigate the molecular mechanisms involved in the progression of LUAD and identify novel therapeutic targets for cancer therapy.

Ubiquitination is a common post-translational modification and is involved in numerous biological processes, such as cell cycle progression, tumorigenesis, and malignant development [4]. Ubiquitination is catalyzed by three enzymes: E1-activating enzymes, E2-conjugating enzymes, and E3-ligating enzymes [5, 6]. Among these enzymes, E2-conjugating enzymes are important mediators that help in the transfer of ubiquitin to a target lysine side chain [7]. The dysregulation of E2-conjugating enzymes contributes to a variety of tumor-promoting processes [8].

As a member of the E2-conjugating enzyme family, UBE2N, commonly known as UBC13, has been demonstrated to play key roles in the progression of various cancers. For example, in melanoma, UBE2N regulates the MEK/FRA1/SOX10 signaling cascade, which is crucial for proliferation, survival, and malignant progression [9]. Another research demonstrated that UBE2N was highly expressed in primary and metastatic breast cancer, and promoted the metastatic spread of breast cancer cells by controlling their lung-colonizing ability [10]. Notably, specific inhibition of UBE2N by a small-molecule inhibitor could induce neuroblastoma cell death [11], highlighting a potential role for UBE2N as a therapeutic target in cancer therapy. However, the functions and mechanisms underlying UBE2N expression in LUAD are still unclear.

As the founder member of the Sp transcription factor family, SP1 contains C2H2-type zinc fingers and can activate or repress transcription of downstream target genes by binding to the GC-rich motifs in the promoter regions of the target genes [12]. SP1 participates in many cellular processes, including differentiation, growth, apoptosis, immune responses, angiogenesis, etc. [13]. It has become increasingly clear that SP1 plays a critical role in cancer progression. Recent evidence showed that SP1 facilitated the progression of colorectal cancer and hepatocellular carcinoma by mediating the TUG1-miR-421-KDM2A-ERK and DUBR/E2F1-CIP2A axis respectively [14, 15]. In addition, another study of gastric cancer indicated that SP1 promoted cancer growth and metastasis by transcriptionally activating the Oncostatin M receptor [16]. Interestingly, SP1 has also been reported to be essential in LUAD progression, including proliferation, migration, invasion, and metastasis [17, 18]. SP1 mediates LUAD progression, but its molecular mechanism remains unidentified.

In this study, a high expression of UBE2N was observed in LUAD patients, and those patients had poor clinical outcomes. Furthermore, inhibition of UBE2N significantly reduced LUAD progression in vitro and in vivo. At the molecular level, we demonstrated that UBE2N was upregulated by the transcription factor SP1 at the transcriptional level, which consequently confers LUAD progression. Moreover, the expression of SP1 and UBE2N in human LUAD samples was strongly correlated. Collectively, our data revealed that the SP1-UBE2N axis might be crucial to the malignant progression of LUAD, providing new therapeutic targets and strategies for the treatment of LUAD.

## Results

### A high level of UBE2N expression in LUAD is associated with a poor prognosis

**Fig. 1:**
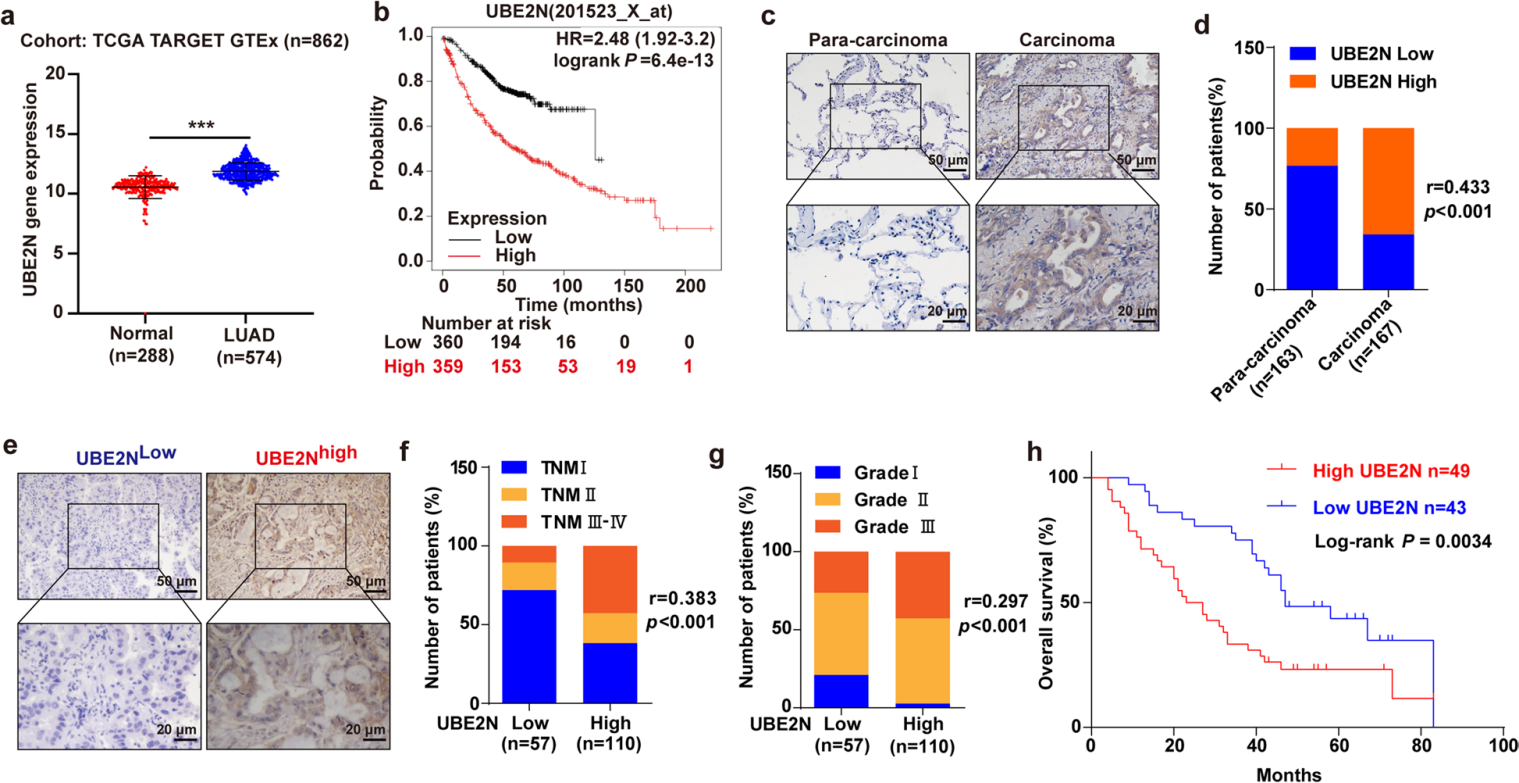
A high level of UBE2N expression in LUAD is associated with a poor prognosis. **a** UBE2N expression was analyzed in LUAD tissues as well as normal lung tissues from the TCGA TARGET GTEx cohort. ***P < 0.001. **b** Relationship between UBE2N expression and overall survival in LUAD patients using KM plotter. **c, d** UBE2N expression was detected using an immunohistochemical technique in LUAD and adjacent normal tissues. *r* = 0.433, *P* < 0.001. **e** Representative IHC images of UBE2N in LUAD tissues. **f**, **g** The expression of UBE2N correlates positively with TNM stage **f** (*r* = 0.383, *P* < 0.001) and pathological grade **g** (*r* = 0.297, *P* < 0.001) in human LUAD samples. **h** High expression of UBE2N is correlated with poor overall survival of LUAD patients. Log-rank *P* = 0.0034

To investigate the function of UBE2N in LUAD, we first analyzed the expression of UBE2N in normal lung and LUAD samples from the Genotype-Tissue Expression (GTEx) and The Cancer Genome Atlas (TCGA) database. The results showed that UBE2N expression was significantly increased in LUAD samples compared to normal lung samples (Fig. 1a). Subsequent analysis from the online Kaplan-Meier Plotter database showed that high UBE2N expression was associated with poor overall survival in patients with LUAD (Fig. 1b). To further demonstrate the above observation, we examined the expression of UBE2N in human LUAD and para-carcinoma tissues by immunohistochemistry. LUAD tissues were much more highly expressed of UBE2N than para-carcinoma tissues, as shown in Fig. 1c, d. We then classified the LUAD subjects into two groups according to UBE2N expression (Fig. 1e). The results showed that UBE2N expression was positively associated with the high TNM stage and advanced pathological grade (Fig. 1f, g). Additionally, survival analysis revealed that LUAD patients with high UBE2N expression were more likely to have shorter overall survival than those with low UBE2N expression (Fig. 1h). Altogether, these results indicated that UBE2N may play an important role in the progression of LUAD.

### Inhibition of UBE2N reduces LUAD growth in vitro and in vivo

**Fig. 2:**
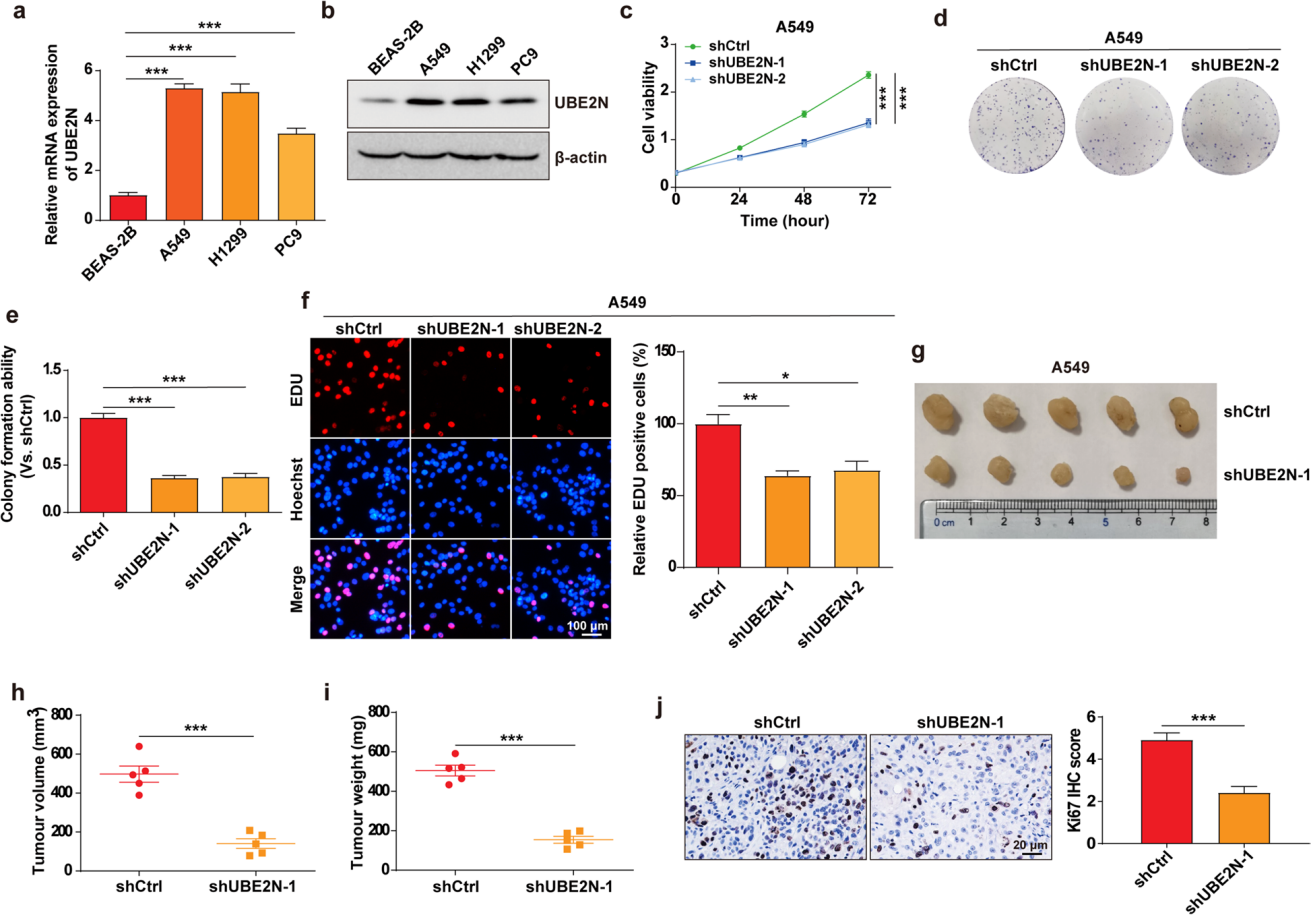
Inhibition of UBE2N reduces LUAD growth in vitro and in vivo. **a**, **b** The UBE2N mRNA (**a**) and protein (**b**) expression in lung/brunch epithelial cells (BEAS-2B) and LUAD cells (A549, H1299, and PC9). ***P < 0.001. **c** Cell proliferation was analyzed by CCK-8 assay at the indicated time points. ***P < 0.001. **d**, **e** Cell proliferation was analyzed by Colony formation assay. ***P < 0.001. **f** EDU incorporation assay was used to analyze cell proliferation. **P < 0.01, *P < 0.05. **g** Images of xenograft LUAD tumor tissues from the shCtrl and shUBE2N groups. **h**, **i** Tumor volume (**h**) and tumor weight (**i**) in shCtrl and shUBE2N A549 xenograft mouse model. ***P < 0.001. **j** IHC images of Ki-67 in tumor sections from shCtrl and shUBE2N A549 xenograft mouse model. ***P < 0.001

To determine whether UBE2N contributes to LUAD development, we examined the expression of UBE2N in LUAD cell lines and normal lung/brunch epithelial cell line BEAS-2B. The results show that UBE2N expression was higher in LUAD cell lines including A549, H1299, and PC9 compared to BEAS-2B (Fig. 2a, b). Thus, A549 and H1299 cells were infected with a lentivirus expressing shRNAs specific for UBE2N (shUBE2N-1 and shUBE2N-2) or a control shRNA (shCtrl). UBE2N depletion by specific shRNAs significantly reduced UBE2N mRNA and protein expression (Supplementary Fig. 1a-d). Then, the CCK-8, colony formation, and EDU incorporation assays were performed to analyze cell proliferation. The results showed that the knockdown of UBE2N significantly reduced cell proliferation in A549 and H1299 cells (Fig. 2c-f and Supplementary Fig. 2a-c).

To further confirm these results, we injected A549 cells stably expressing control shRNA, UBE2N shRNA into BALB/c nude mice, and monitored tumor growth in vivo. Xenograft model experiment indicated that UBE2N depletion drastically reduced tumor growth, tumor size, and tumor weight (Fig. 2g-i). Immunohistochemistry staining also showed that the expression of proliferation marker Ki67 was significantly downregulated in UBE2N knockdown group (Fig. 2j). Together, these results demonstrate that UBE2N plays a key role in LUAD growth.

### SP1 transcriptionally regulates UBE2N expression in LUAD cells

**Fig. 3:**
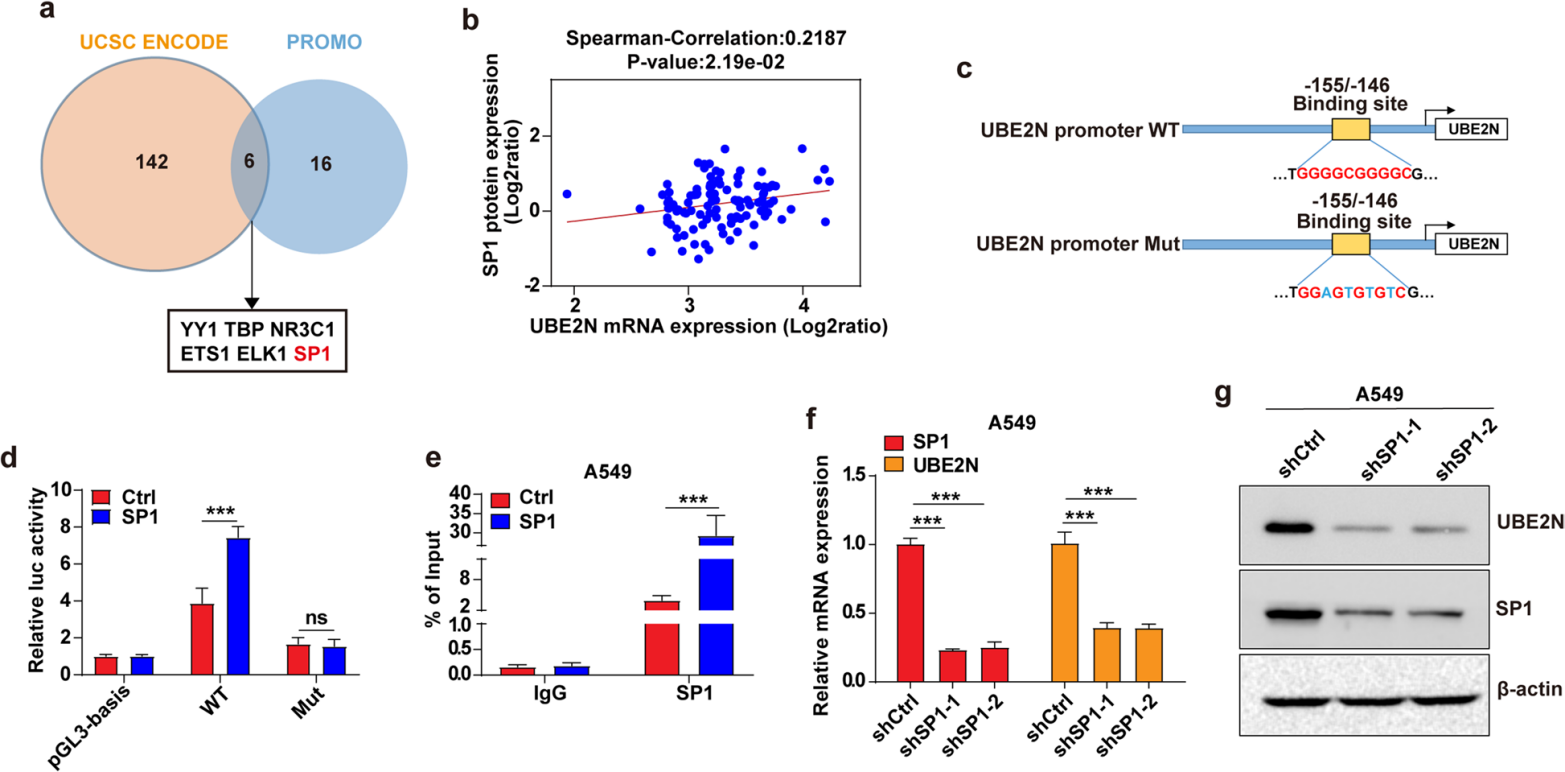
SP1 regulates UBE2N transcription in LUAD cells. **a** The potential TFs were predicted using the UCSC ENCODE and PROMO public prediction websites. Potential candidates included six TFs. **b** The correlation between SP1 protein expression and UBE2N mRNA expression in LUAD was analyzed by the LinkedOmics platform. **c** A putative binding element of SP1 was shown in the promoter region of UBE2N. **d** Dual-luciferase reporter assays were performed by co-transfection of the pGL3‑basic, pGL3-UBE2N-WT or pGL3-UBE2N-MUT promoter vector and pLV-Ctrl or pLV-SP1 expression vector. ***P < 0.001. **e** The ChIP-qPCR assay showed that SP1 overexpression significantly increased its recruitment to the UBE2N promoter. ***P < 0.001. **f**, **g** Expression of UBE2N and SP1 mRNA (**f**) and protein (**g**) in shCtrl/A549 and shSP1/A549 cells. ***P < 0.001

To investigate how UBE2N was regulated in LUAD, UCSC ENCODE and PROMO public websites were used to predict transcription factors involved in UBE2N transcription. As shown in Fig. 3a, six TFs were screened out. Notably, the correlation between SP1 protein and UBE2N mRNA expression levels was much higher as compared with the five other TFs in LUAD (Fig. 3b and Supplementary Fig. 3a-e), indicating that SP1 might regulate UBE2N expression. SP1 belongs to the zinc finger transcription factors, which regulate gene transcription by binding to their promoter consensus motifs [19]. Thus, the PROMO platform was used to predict potential SP1 binding sites in the UBE2N gene promoter (2 kb). As shown in Fig. 3c, the UBE2N promoter region contains a major putative binding site (BS). Then, to determine whether SP1 regulates UBE2N transcription, as shown in Fig. 3c, the luciferase assay vectors containing wild-type or mutated SP1 binding site sequences of UBE2N promoter were transiently co-transfected with SP1 overexpression vector into A549 cells. The luciferase assay showed that overexpression of SP1 increased the promoter activity of UBE2N while mutated BS reduced luciferase activity (Fig. 3d). Furthermore, ChIP-qPCR revealed that in comparison with the control group, SP1 overexpression enhanced its recruitment to the UBE2N promoter (Fig. 3e). Moreover, qPCR and WB results showed that knockdown of SP1 significantly decreased UBE2N expression in A549 and H1299 cells (Fig. 3f, g and Supplementary Fig. 3f, g). Altogether, these results indicated that BS in the UBE2N promoter is crucial for SP1 transcriptionally regulated UBE2N expression in LUAD cells.

### Highly expressed SP1 in LUAD is correlated with worse clinical outcome

**Fig. 4:**
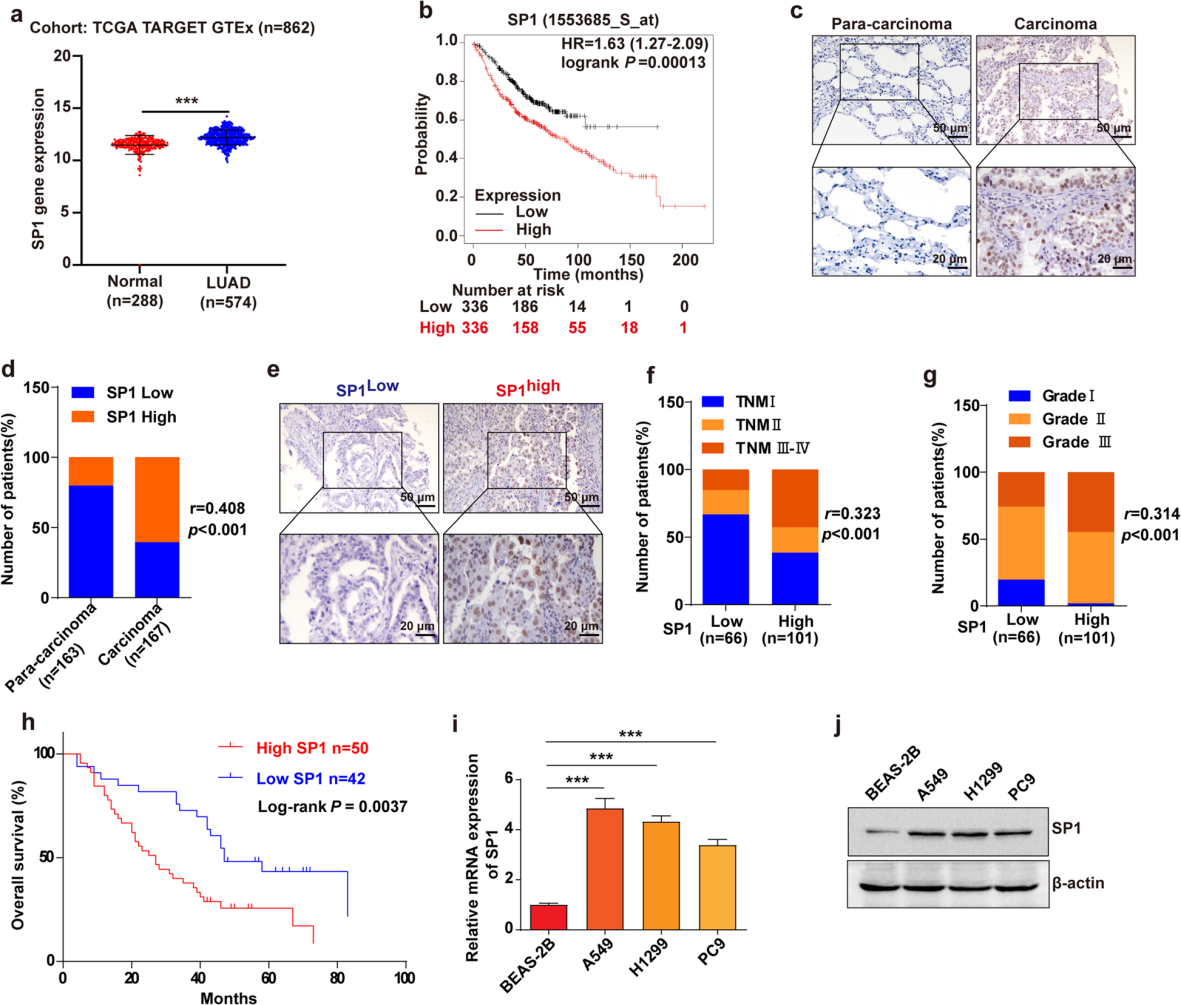
Highly expressed SP1 in LUAD is correlated with worse clinical outcomes. **a** In the TCGA TARGET GTEx cohort, SP1 expression was analyzed in LUAD tissues and normal lung tissues. ***P < 0.001. **b** Relationship between SP1 expression and overall survival in LUAD patients using KM plotter. **c**, **d** SP1 expression was detected using an immunohistochemical technique in LUAD and adjacent normal tissues. *r* = 0.408, *P* < 0.001. **e** Representative IHC images of SP1 in LUAD tissues. **f**, **g** The expression of SP1 correlates positively with TNM stage **f** (*r* = 0.323, *P* < 0.001) and pathological grade **g** (*r* = 0.314, *P* < 0.001) in human LUAD samples. **h** High expression of SP1 is correlated with poor overall survival of LUAD patients. Log-rank *P* = 0.0037. **i**, **j** The SP1 mRNA (**i**) and protein (**j**) expression in lung/brunch epithelial cells (BEAS-2B) and LUAD cells (A549, H1299, and PC9). ***P < 0.001

Previous studies have shown that multiple cancers were associated with overexpression of SP1, such as gastric cancer [20], pancreatic ductal adenocarcinoma [21], and lung cancer [22]. However, the expression and function of SP1 in LUAD remain unclear, and need further investigation. Thus, we analyzed SP1 expression in normal lung and LUAD tissues using the TCGA dataset and GTEx dataset. As shown in Fig. 4a, a higher level of SP1 expression was observed in LUAD samples than in normal lung samples. We further investigated the correlation between SP1 expression and LUAD patients’ prognosis using the online Kaplan-Meier Plotter database. The results showed that high SP1 expression in LUAD patients was associated with shorter survival than low SP1 expression in these patients (Fig. 4b). Furthermore, we performed immunohistochemistry to detect the expression of SP1 in LUAD tissues and para-carcinoma tissues. As shown in Fig. 4c, d, SP1 was significantly increased in LUAD tissues as compared to para-carcinoma tissues. Then we classified the LUAD subjects into two groups according to the expression of SP1 (Fig. 4e). The results showed that SP1 expression was positively correlated with high TNM stage and advanced pathological grade (Fig. 4f, g). And the survival analysis showed that elevated levels of SP1 were associated with poorer overall survival in LUAD patients (Fig. 4h). Furthermore, we determined the expression of SP1 in LUAD cell lines and normal lung/brunch epithelial cell line BEAS-2B. According to the results, SP1 was significantly more expressed in LUAD cell lines A549, H1299, and PC9 than in BEAS-2B (Fig. 4i, j). Together, these observations suggested that SP1 was highly expressed in LUAD and was associated with poor clinical outcomes.

### UBE2N antagonizes the effect of SP1 knockdown in regulating LUAD growth

**Fig. 5:**
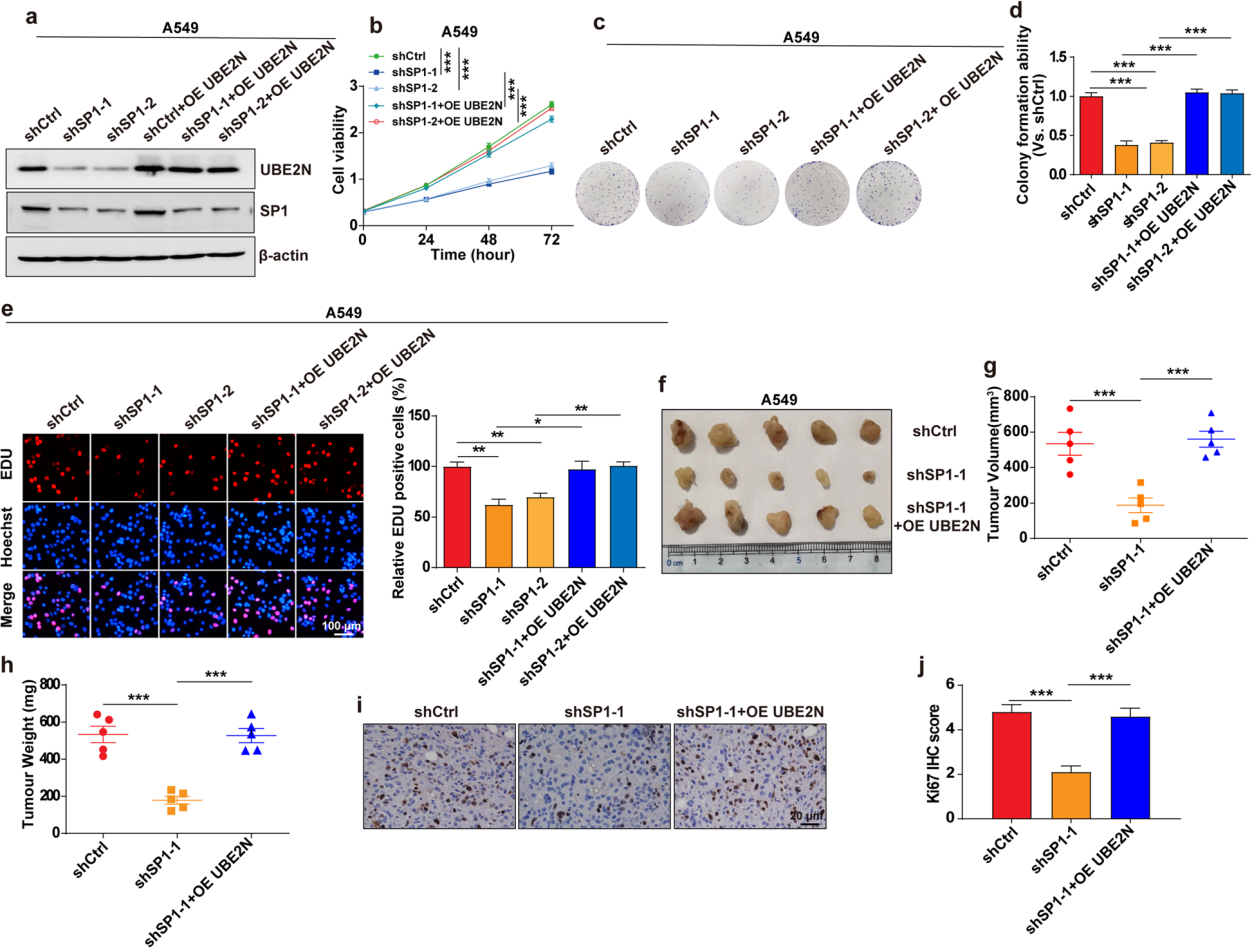
UBE2N antagonizes the effect of SP1 knockdown in regulating LUAD growth. **a** SP1 and UBE2N protein expression in shCtrl/A549 and shSP1/A549 cells with or without exogenous overexpression of UBE2N. **b**-**e** CCK-8 (**b**), Colony formation (**c**, **d**), and EDU incorporation assay (**e**) in SP1-interfered A549 cells with or without exogenous overexpression of UBE2N. *P < 0.05, **P < 0.01, ***P < 0.001. **f** Images of xenograft LUAD tumor tissues from the BALB/c nude mice that were injected with SP1-interfered A549 cells with or without exogenous overexpression of UBE2N. **g**, **h** Tumor volume (**g**) and tumor weight (**h**) in SP1-interfered A549 cells with or without exogenous overexpression of the UBE2N xenograft model. ***P < 0.001. **i**, **j** IHC images of Ki-67 in tumor sections from xenograft model that were injected with SP1-interfered A549 cells with or without exogenous overexpression of UBE2N. ***P < 0.001

To explore whether UBE2N is involved in SP1-mediated LUAD growth, UBE2N was overexpressed based on SP1 knockdown in LUAD cells (Fig. 5a and Supplementary Fig. 4a). Then, the CCK-8, colony formation, and EDU incorporation assays revealed that knockdown of SP1 markedly suppressed LUAD cells proliferation; however, there was a significant reduction in this effect with the exogenous overexpression UBE2N (Fig. 5b-e and Supplementary Fig. 4b-d). We further investigated the effects of SP1 deletion on LUAD growth in vivo. Consequently, BALB/c nude mice were subcutaneously injected with SP1-depleted cells with or without exogenous overexpression of UBE2N and then monitored tumor growth in vivo. As shown in Fig. 5f-h, SP1 knockdown significantly suppressed tumor growth, tumor size, and tumor weight. However, this effect was reduced in mice with exogenous overexpression of UBE2N, indicating that SP1 deletion resulted in reduced tumor growth by regulating UBE2N in vivo. Furthermore, immunohistochemistry staining showed that the expression of proliferation marker Ki67 was significantly reduced in SP1 knockdown group (Fig. 5i, j), while this effect was abolished in UBE2N overexpressing group. Thus, SP1 regulates LUAD growth through UBE2N.

### The expression of UBE2N correlates positively with SP1 in LUAD patients

**Fig. 6:**
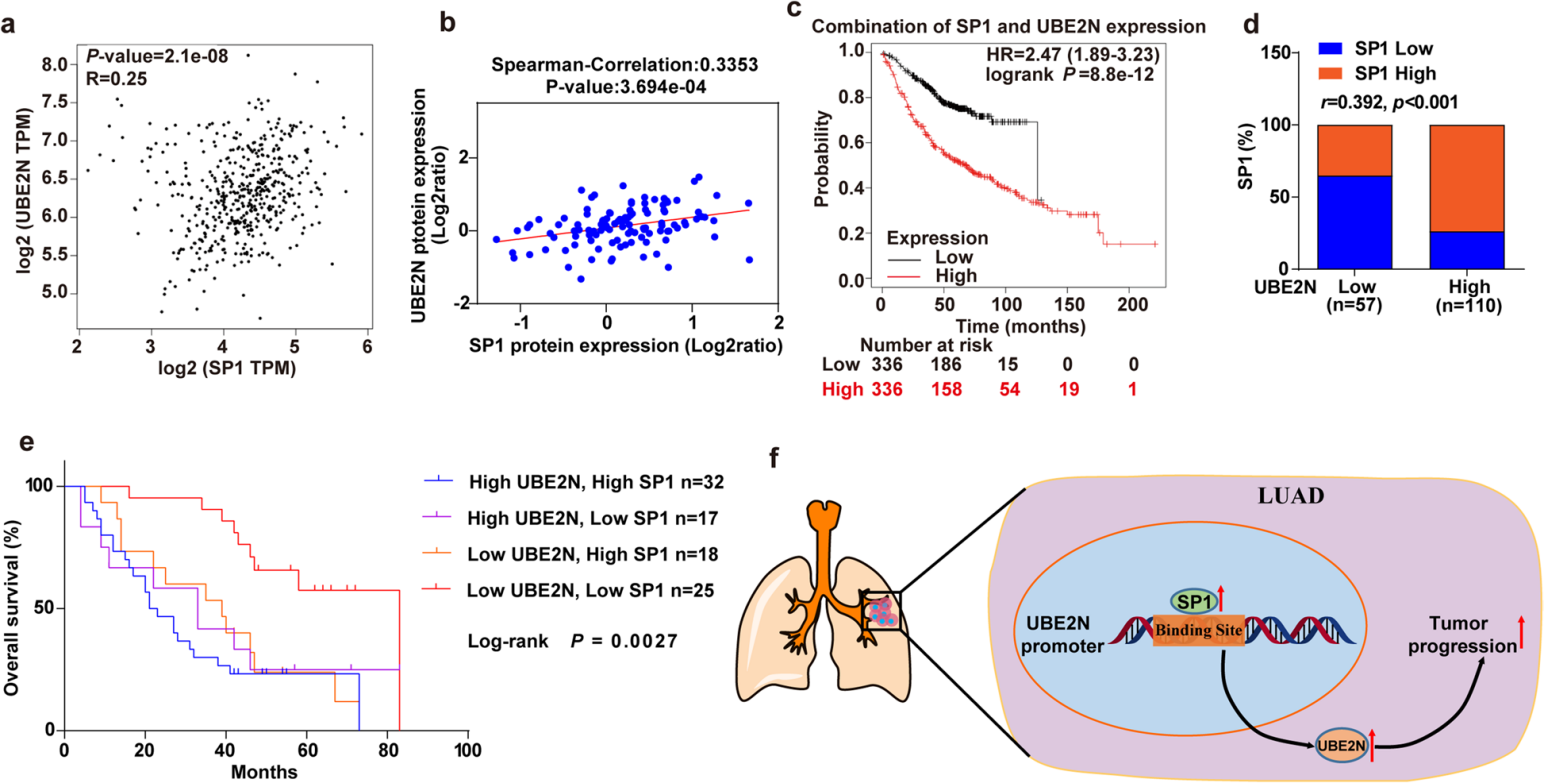
The expression of UBE2N is positively correlated with SP1 in human LUAD patients. **a** The correlation between SP1 mRNA expression and UBE2N mRNA expression in LUAD was analyzed by the GEPIA platform. **b** The correlation between SP1 protein expression and UBE2N protein expression in LUAD was analyzed by the LinkedOmics platform. **c** Relationship between combined SP1 and UBE2N expression and overall survival in LUAD patients using KM plotter. **d** The expression of SP1 and UBE2N are positively correlated in LUAD samples. **e** Kaplan–Meier curves show that the overall survival of patients with high SP1 and UBE2N expression in LUAD tumors was shorter than that of those with low SP1 and UBE2N expression Log-rank *P* = 0.0027. **f** Schematic diagram of the SP1-UBE2N signaling axis in LUAD progression (drawn using ChemDraw software)

To confirm the correlations between SP1 and UBE2N, we examined the SP1 mRNA and UBE2N mRNA expression in LUAD samples derived from the TCGA database using the online tool GEPIA. The results showed that there is a significant positive correlation between SP1 mRNA and UBE2N mRNA expression (Fig. 6a). Moreover, correlation analysis using the online tool LinkedOmics indicated that SP1 protein expression was positively correlated with UBE2N protein in LUAD samples (Fig. 6b). Furthermore, the survival analysis using online tool KM-plotter showed that the highly combined expression of SP1 and UBE2N was associated with poor overall survival of LUAD patients (Fig. 6c). To further demonstrate the above observation, we performed the immunohistochemistry to detect the expression of SP1 and UBE2N in LUAD tissues, and classified the LUAD subjects into two groups according to the expression of SP1 and UBE2N (Figs. 1e and 4e). SP1 and UBE2N expression showed a strong positive correlation (Fig. 6d). Importantly, the overall survival of patients with high SP1 and UBE2N expression in LUAD tumors was shorter than that of those with low SP1 and UBE2N expression (Fig. 6e), indicating that the SP1-UBE2N signaling pathway promotes the progression of LUAD and could be a potential therapeutic target.

## Discussion

LUAD is the leading cause of cancer-related death worldwide [23], and identification of the molecular mechanisms underlying LUAD development and progression is urgently needed. In this study, we demonstrated that UBE2N was overexpressed in LUAD. Moreover, inhibition of UBE2N could reduce LUAD progression. Mechanistically, UBE2N was upregulated by the SP1 transcription factor, which in turn contributed to the development of LUAD. Collectively, our study demonstrated that the SP1-UBE2N axis was a key regulatory mechanism of LUAD progression and provided an important basis for the clinical treatment of advanced lung cancer (Fig. 6f).

UBE2N is a member of the E2-conjugating enzymes family. Members of the E2-conjugating enzymes family play an important role in many types of cancers [24]. For example, there has been an increase in UBE2C levels in various types of cancer (e.g. breast cancer, esophageal squamous cell carcinoma, and lung cancer), and its overexpression correlates with poor prognosis in patients [25–27]. Furthermore, another research found that primary lung cancer tissue and metastatic nodules exhibited a high level of UBE2I expression, which in turn facilitated cancer progression [28]. In this study, we found that UBE2N, another member of the E2-conjugating enzymes family, was significantly increased in LUAD and was negatively correlated with the overall survival of patients, indicating that UBE2N may be a potential prognostic indicator for LUAD patients. Furthermore, our studies revealed that specific inhibition of UBE2N could reduce LUAD progression. And according to a study that the specific inhibitor of E2-conjugating enzyme Ubc13-Uev1A could impede the progression of diffuse large B-cell lymphoma [29], further research on UBE2N inhibitors could provide an effective method to inhibit the progression of LUAD.

Although UBE2N is overexpressed in many types of cancer, little is known about the mechanisms responsible for that overexpression. Song et al. showed that miR-590-3p inhibited the expression of UBE2N through the direct binding of its 3′-UTR in cervical carcinoma cells [30]. Furthermore, UBE2N expression is tightly regulated by deubiquitinase OTUB1 and the zinc finger protein A20 at the posttranslational level [31, 32]. In addition, another study showed that STAT3 could repress UBE2N transcription by binding to the STATx site in its promoter [33]. Here, our study provided further insight into the complex regulation of UBE2N expression in cancer progression by demonstrating that transcription factor SP1 promotes UBE2N expression at the transcriptional level via binding to the promoter of UBE2N. SP1 is the founding member of the SP transcription factor family, which is overexpressed in several cancers [12]. In this regard, our results found that the expression of SP1 was upregulated in LUAD, and its upregulation could induce LUAD progression by promoting UBE2N expression. In addition, SP1 expression and UBE2N expression in LUAD exhibited a significant positive correlation, and the highly combined expression of SP1 and UBE2N was associated with poor overall survival of LUAD patients. Thus, our research demonstrated that the SP1-UBE2N axis was one of the important mechanisms of LUAD progression, which could be an effective therapeutic target for the treatment of LUAD. Although this study has demonstrated SP1 promotes LUAD progression by increasing UBE2N expression, it is unclear how exactly UBE2N promotes LUAD progression. Hence, further research needs to be done to determine the potential mechanisms by which UBE2N regulates the progression of LUAD.

## Materials and methods

### Database analysis

The UBE2N and SP1 expression data of the Genotype-Tissue Expression (GTEx) and The Cancer Genome Atlas (TCGA) samples were downloaded through the UCSC Xena platform [34] and manually graphed in GraphPad Prism software. Survival analyses were performed using the Kaplan-Meier Plotter (KM plotter) database [35]. UCSC ENCODE [36] and PROMO [37, 38] platforms were used to predict transcription factors (TFs) involved in UBE2N transcription. PROMO platform was used to identify transcription factor binding sites for SP1 within the human UBE2N promoter. The correlation between UBE2N mRNA and TFs (YY1, TBP, NR3C1, ETS1, ELK1, and SP1) protein expression was analyzed by the LinkedOmics platform [39]. The correlation between UBE2N protein and SP1 protein expression was also analyzed by the LinkedOmics platform [39]. The correlation between SP1 mRNA and UBE2N mRNA expressions was analyzed using the Gene Expression Profiling Interactive Analysis (GEPIA) platform [40].

### Tissue microarray and IHC staining

A total of 75 cases of human LUAD and 75 cases of para-carcinoma tissues were obtained from SHANGHAI OUTDO BIOTECH CO., LTD., China. (Cat No.: HLugA150CS03), and 92 cases of human LUAD and 88 cases of para-carcinoma tissues with overall survival rates were obtained from SHANGHAI OUTDO BIOTECH CO., LTD., China. (Cat No.: HLugA180Su04). The IHC staining and evaluation were performed as previously described [41], using 1:100 dilution of an anti-UBE2N antibody (Proteintech, 10243-1-AP) and 1:200 dilution of an anti-SP1 antibody (Proteintech, 21962-1-AP).

### Cell culture

BEAS-2B, A549, H1299, and PC9 cell lines were purchased from the ATCC, cultured in RPMI1640 medium (Biological Industries) with 10% fetal bovine serum (Biological Industries) and 1% penicillin-streptomycin (Gibco Life Technologies) at 37 °C in 5% CO_2_.

### RNA extraction and quantitative RT-PCR

Trizol reagent (Invitrogen) was used to isolate total RNA from cells. Subsequently, reverse transcription was performed with TransScript First-Strand cDNA Synthesis SuperMix (TransGen Biotech). Then, the TransStart Green Q-PCR SuperMix (TransGen Biotech) was used to detect UBE2N and SP1 expression. To normalize, GAPDH was used. Primer sequences are provided in Supplementary Table 1.

### Western blotting

Western blotting was performed as described previously [42], using 1:1000 dilution of an anti-UBE2N antibody (Proteintech, 10243-1-AP), 1:1000 dilution of an anti-SP1 antibody (Proteintech, 21962-1-AP) and 1:1000 dilution of an anti-β actin antibody (Proteintech, 20536-1-AP).

### Lentiviral knockdown and expression systems

We annealed shRNAs for UBE2N and SP1 and cloned them into the pLV-H1-EF1α-puro vector (Biosettia). The human cDNA fragment encoding UBE2N or SP1 was cloned into the pLV-EF1-MCS-IRES-Bsd vector (Biosettia). Lentiviral vectors and packaging plasmids were transfected into HEK293T cells via Lipofectamine 2000 reagent (Invitrogen) to generate lentiviruses. Primer sequences are provided in Supplementary Table 1.

### Cell proliferation assay

Lung cancer cell proliferation was measured through CCK-8, colony formation, and EDU incorporation assays, which were performed as described previously [43].

### Tumor xenograft experiments

All the experiments were approved by Ethics Committee at the Jiangxi provincial People’s Hospital (Approval numbers 2021-017). Six-week-old BALB/c nude mice were obtained from Beijing Vital River Laboratory Animal Technology Co., Ltd. A549 cells (2 × 10^6^) were subcutaneously injected into the right flank of BALB/c nude mice. 10 days after inoculation, tumor sizes were measured every 3 days. The mice were sacrificed after 20 days, and the tumors were harvested, weighed, and photographed. Then, the tumors were dehydrated and embedded in paraffin. Finally, sections were cut at a thickness of 4 μm for IHC staining using 1:100 dilution of an anti-Ki67 antibody (Abcam, ab15580).

### Luciferase assay

A pGL3basic vector (Promega) was used to clone the human UBE2N-WT and UBE2N-MUT promoters. In Supplementary Tables 1, primer sequences are provided. Lipofectamine 2000 reagent (Invitrogen) was used to co-transfect cells with pGL3basic, pGL3-UBE2N-WT, or pGL3-UBE2N-MUT promoter vectors as well as pLV-Ctrl or pLV-SP1 expression vectors. Following the manufacturer’s instructions, we measured luciferase activity after 48 h using a dual-luciferase reporter assay system (Promega).

### ChIP-qPCR

Following the manufacturer’s instructions, ChIP assays were performed using the EZ-ChIP kit (Millipore). Briefly, a 1% formaldehyde solution was applied to cells for 10 min at room temperature followed by 125mM glycine quenching. Chromatin extracts containing DNA fragments were immunoprecipitated using an anti-SP1 antibody (Proteintech, 21962-1-AP). The ChIP-enriched DNA was then used for quantitative PCR with the specific primers listed in Supplementary Table 1.

### Statistical analysis

Statistical analysis was performed using SPSS 17.0 and GraphPad Prism software. We present the data as mean ± SEM. The gene correlation was analyzed in tissue samples using the Spearman correlation test. Student’s t-test was used for the statistical analysis of two groups. The survival analysis was conducted using the Kaplan-Meier and log-rank tests. A *P* value less than 0.05 was considered significant.

## Supplementary Information

**Additional file 1: Supplementary Figure 1.** (a-d) qRT-PCR (a, b) and Western blot (c, d) analysis of UBE2N knockdown in A549 and H1299 cells. ***P < 0.001. **Supplementary Figure 2.** (a) Cell proliferation was analyzed by CCK-8 assay at the indicated time points. ***P < 0.001. (b) Cell proliferation was analyzed by Colony formation assay. ***P < 0.001. (c) Cell proliferation wasanalyzed by EDU incorporation assay. **P< 0.01. **Supplementary Figure 3.** (a-e) The correlation between YY1 (a), TBP (b), NR3C1 (c), ETS1 (d), and ELK1 (e) protein expression and UBE2N mRNA expression in LUAD was analyzed by LinkedOmics platform. (f, g) The mRNA (f) and protein (g) expression of UBE2N and SP1 in shCtrl/H1299 and shSP1/H1299 cells. ***P < 0.001. **Supplementary Figure 4.** (a) The protein expression of SP1 and UBE2N in shCtrl/H1299 and shSP1/H1299 cells in the presence or absence of overexpressed UBE2N. (b-d) CCK-8 assay (b), Colony formation assay (c) and EDU incorporation assay (d) in SP1-interfered H1299 cells in the presence or absence of rescued UBE2N expression. ***P < 0.001, **P < 0.01, *P < 0.05. **Supplementary Table 1.** Primers used in this study. 

## Data Availability

The data of this study are available from the corresponding author upon reasonable request.
