# Supplementary material for: SP1 transcriptionally regulates UBE2N expression to promote lung adenocarcinoma progression
Source: Mol Biomed. 2023 Mar 25;4:7. doi: 10.1186/s43556-023-00118-2 (PMC10039148; doi:10.1186/s43556-023-00118-2)
Supplement: Supplementary file 1 — Additional file 1: Supplementary Figure 1. (a-d) qRT-PCR (a, b) and Western blot (c, d) analysis of UBE2N knockdown in A549 and H1299 cells. ***P < 0.001. Supplementary Figure 2. (a) Cell proliferation was analyzed by CCK-8 assay at the indicated time points. ***P < 0.001. (b) Cell proliferation was analyzed by Colony formation assay. ***P < 0.001. (c) Cell proliferation wasanalyzed by EDU incorporation assay. **P< 0.01. Supplementary Figure 3. (a-e) The correlation between YY1 (a), TBP (b), NR3C1 (c), ETS1 (d), and ELK1 (e) protein expression and UBE2N mRNA expression in LUAD was analyzed by LinkedOmics platform. (f, g) The mRNA (f) and protein (g) expression of UBE2N and SP1 in shCtrl/H1299 and shSP1/H1299 cells. ***P < 0.001. Supplementary Figure 4. (a) The protein expression of SP1 and UBE2N in shCtrl/H1299 and shSP1/H1299 cells in the presence or absence of overexpressed UBE2N. (b-d) CCK-8 assay (b), Colony formation assay (c) and EDU incorporation assay (d) in SP1-interfered H1299 cells in the presence or absence of rescued UBE2N expression. ***P < 0.001, **P < 0.01, *P < 0.05. Supplementary Table 1. Primers used in this study. [file 43556_2023_118_MOESM1_ESM.docx]

Supplementary Information for

SP1 transcriptionally regulates UBE2N expression to promote lung adenocarcinoma progression

Jianjun Li^1,2,3,#^, Chunchun Qi^4,#^, Shanshan Shao^5,#^, Yanru Chen^6^, Zimei Peng^6^, Qinglin Shen^6,7,*^, Zhen Zhang^6,*^

^1^Department of Pulmonary and Critical Care Medicine, The First Affiliated Hospital of Soochow University, 215006 Suzhou, China

^2^Suzhou Key Laboratory for Respiratory Diseases, 215006 Suzhou, China

^3^Institute of Respiratory Diseases, Soochow University, 215006 Suzhou, China

^4^Medical College of Nankai University, Tianjin 300071, China

^5^Nanjing University of Chinese Medicine, Nanjing 210023, China;

^6^Institute of Clinical Medicine, Jiangxi provincial People's Hospital, The First Affiliated Hospital of Nanchang Medical College, Nanchang 330006, Jiangxi, China

^7^Department of Oncology, Jiangxi provincial People's Hospital, The First Affiliated Hospital of Nanchang Medical College, Nanchang 330006, Jiangxi, China

^#^These authors contribute equally to the work.

*Corresponding author: Zhen Zhang

Jiangxi provincial People's Hospital, The First Affiliated Hospital of Nanchang Medical College, 152 Aiguo Road, Nanchang 330006, Jiangxi, China

Tel: +86-791-86896246

E-mail: [nkzhenzhang@163.com](mailto:nkzhenzhang@163.com)

Qinglin Shen

Jiangxi provincial People's Hospital, The First Affiliated Hospital of Nanchang Medical College, 152 Aiguo Road, Nanchang 330006, Jiangxi, China

Tel: +86-791-86896246

E-mail: qinglinshen@whu.edu.cn

**This file includes:** Supplementary Fig. S1-4 and Supplementary Tables S1.

**Fig. S1.**

**
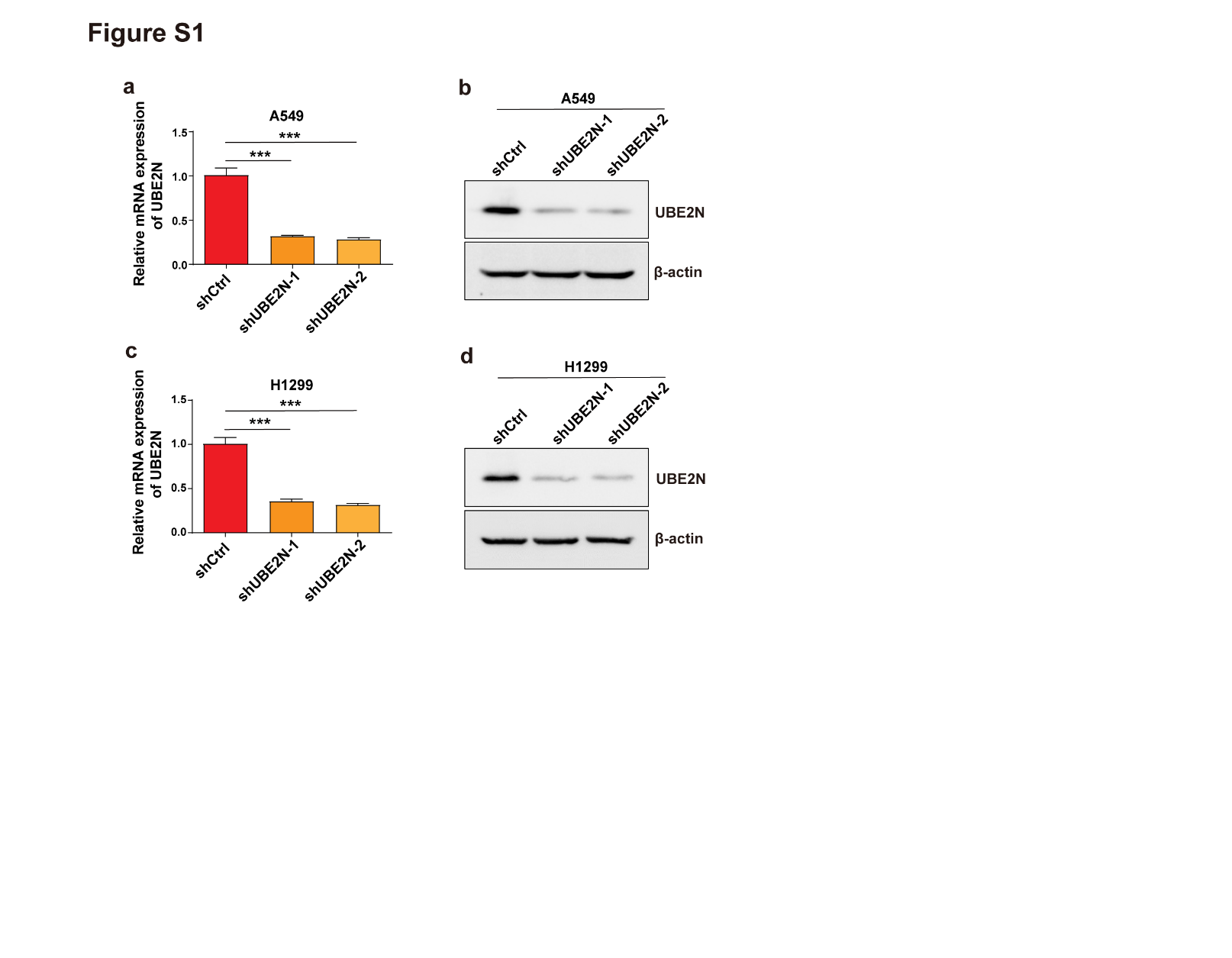
**

**Supplementary Figure 1. (a-d)** qRT-PCR (a, b) and Western blot (c, d) analysis of UBE2N knockdown in A549 and H1299 cells. ****P* < 0.001.

**Fig. S2.**


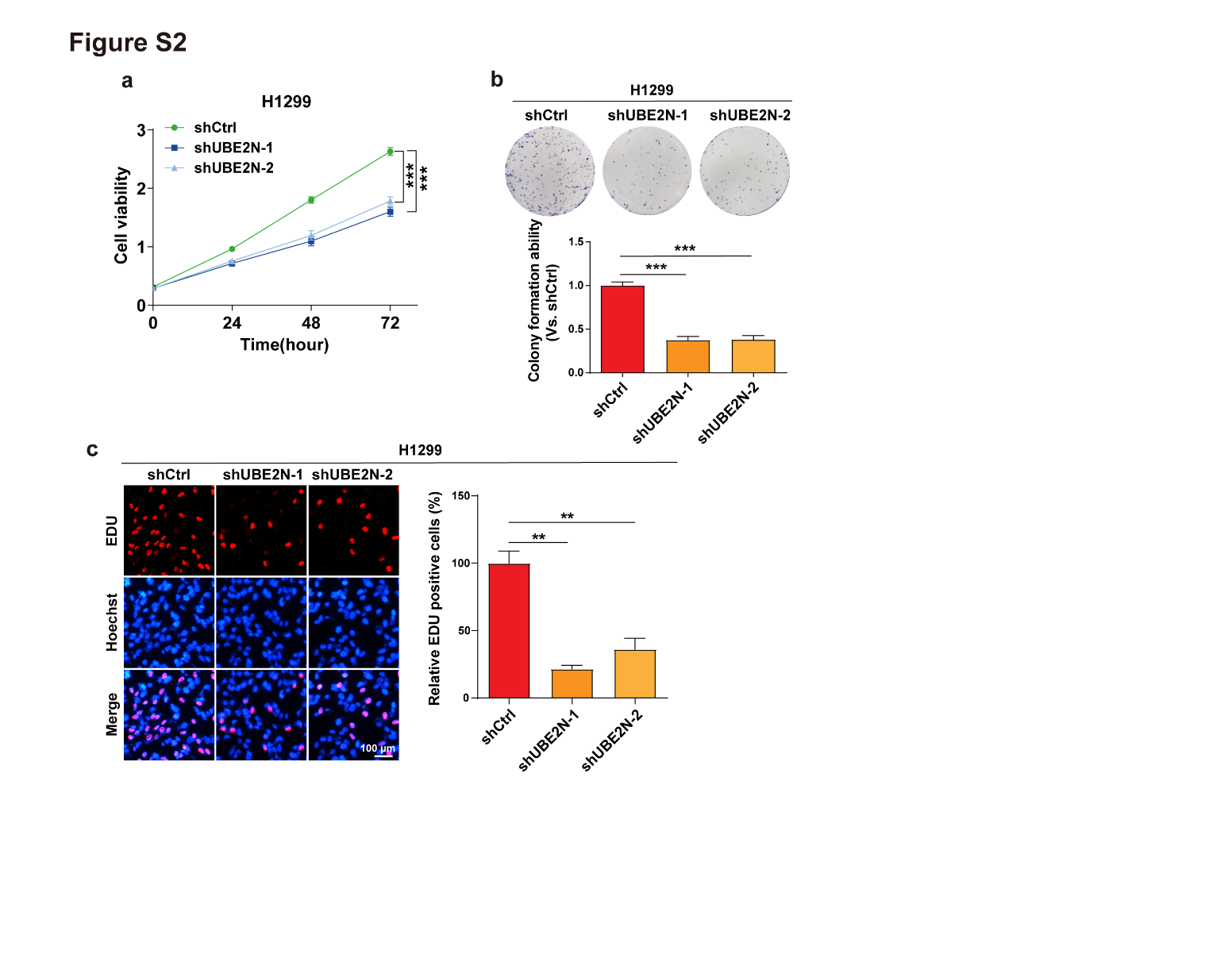


**Supplementary Figure 2.** **(a)** Cell proliferation was analyzed by CCK-8 assay at the indicated time points. ****P* < 0.001. **(b)** Cell proliferation was analyzed by Colony formation assay. ****P* < 0.001. **(c)** Cell proliferation was analyzed by EDU incorporation assay. ***P* < 0.01.

**Fig. S3.**


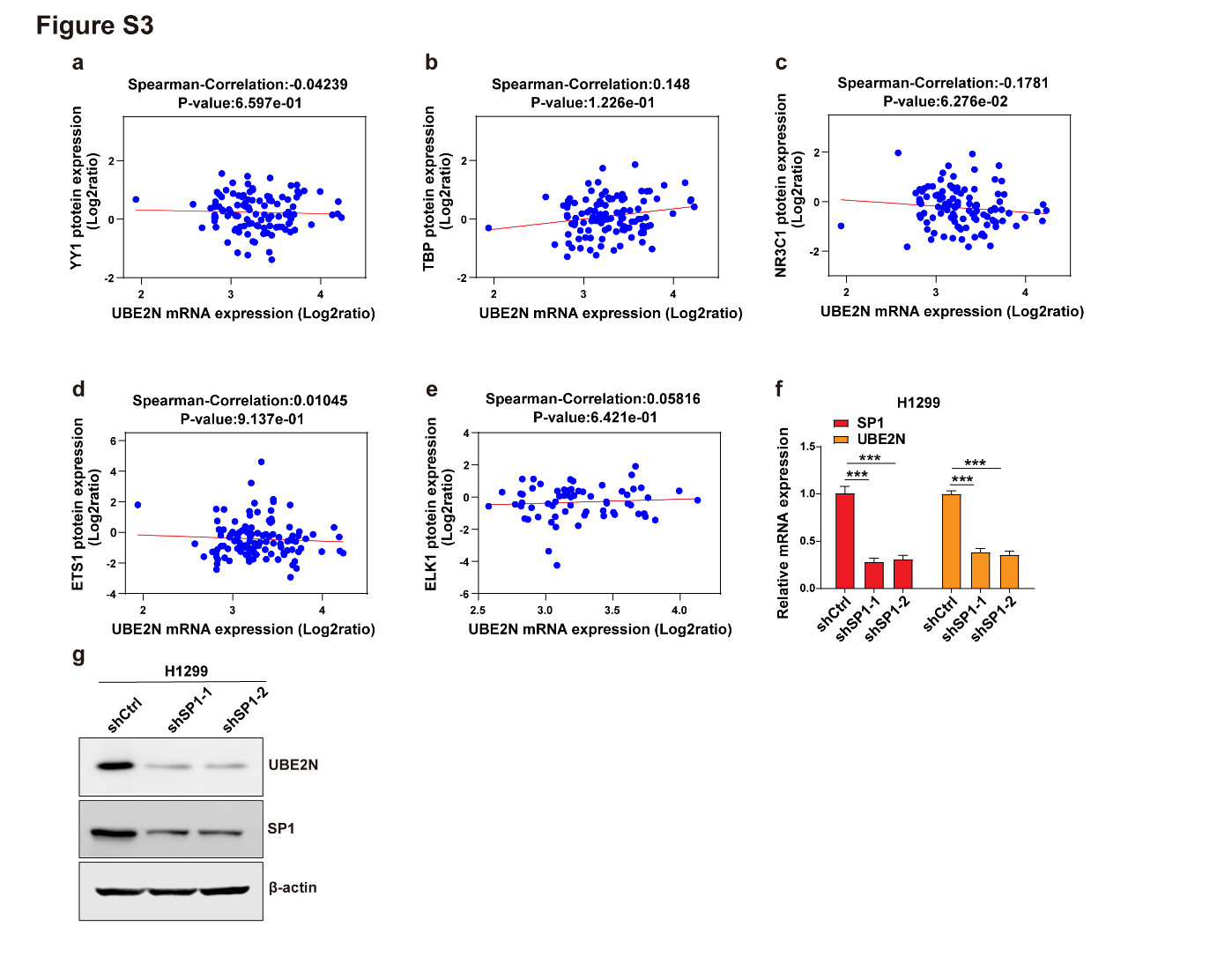


**Supplementary Figure 3.** **(a-e)** The correlation between YY1 (a), TBP (b), NR3C1 (c), ETS1 (d), and ELK1 (e) protein expression and UBE2N mRNA expression in LUAD was analyzed by LinkedOmics platform. **(f, g)** The mRNA (f) and protein (g) expression of UBE2N and SP1 in shCtrl/H1299 and shSP1/H1299 cells. ****P* < 0.001.

**Fig. S4.**


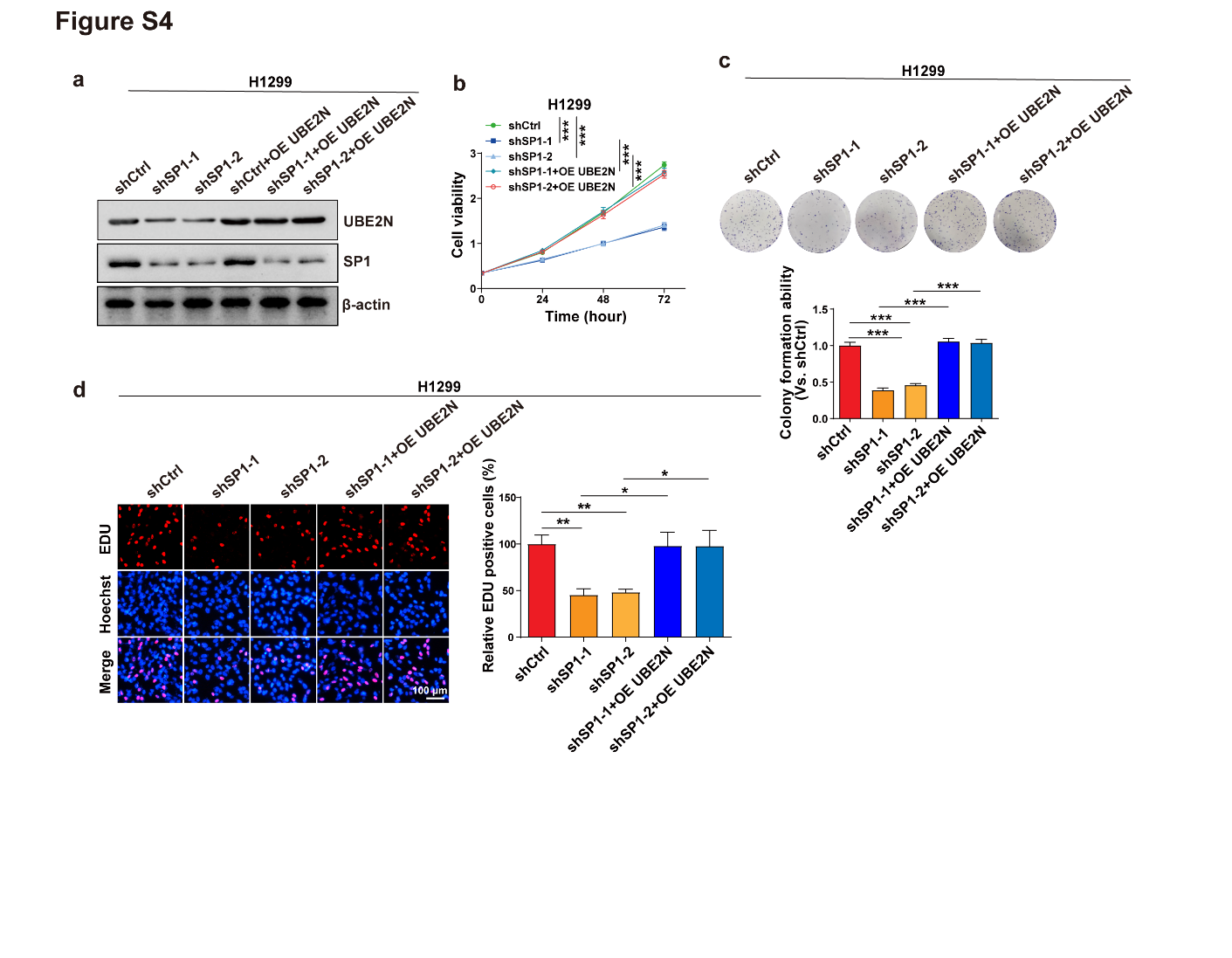


**Supplementary Figure 4. (a)** The protein expression of SP1 and UBE2N in shCtrl/H1299 and shSP1/H1299 cells in the presence or absence of overexpressed UBE2N. **(b-d)** CCK-8 assay **(b)**, Colony formation assay **(c)** and EDU incorporation assay **(d)** in SP1-interfered H1299 cells in the presence or absence of rescued UBE2N expression. ****P* < 0.001, ***P* < 0.01, **P* < 0.05.

**Supplementary Table 1. Primers used in this study.**

| Construction of SP1 expression vector | |
| --- | --- |
| SP1 forward | 5’- ATGAGCGACCAAGATCACTC ‑3’ |
| SP1 reverse | 5’- TCAGAAGCCATTGCCACTGATAT ‑3’ |
| Construction of UBE2N expression vector | |
| UBE2N forward | 5’- ATGGCCGGGCTGCCCCGCAG ‑3’ |
| UBE2N reverse | 5’- TTAAATATTATTCATGGCATATAGCC ‑3’ |
| Construction of UBE2N promoter | |
| UBE2N-WT forward | 5’- CCACGAGGCCATGTGATTT ‑3’ |
| UBE2N-WT reverse | 5’- GGAAGTCCCGGGCTCCACTT ‑3’ |
| Site-Directed Mutagenesis of UBE2N promoter | |
| UBE2N-Mut forward | 5’- GGCAGCGGTTGGTGAGAGGCGCACGGG ‑3’ |
| UBE2N-Mut reverse | 5’- CCCGTGCGCCTCTCACCAACCGCTGCC ‑3’ |
| Construction of shRNA vector | |
| shSP1-1 | 5’- CTCCAAGGCCTGGCTAATAAT‑3’ |
| shSP1-2 | 5’- GCTGGTGGTGATGGAATACAT ‑3’ |
| shUBE2N-1 | 5’- CCTTCCAGAAGAATACCCAAT ‑3 |
| shUBE2N-2 | 5’- AGACAAGTTGGGAAGAATATG ‑3’ |
| Quantitative RT-PCR | |
| SP1 forward | 5’- AGTTCCAGACCGTTGATGGG -3’ |
| SP1 reverse | 5’- GTTTGCACCTGGTATGATCTGT -3’ |
| UBE2N forward | 5’- CCAGAAGAATACCCAATGGCAG -3’ |
| UBE2N reverse | 5’- GCTGGGGACCACTTATCTTTCA -3’ |
| GAPDH forward | 5’- GGAGCGAGATCCCTCCAAAAT -3’ |
| GAPDH reverse | 5’- GGCTGTTGTCATACTTCTCATGG -3’ |
| CHIP-qPCR | |
| Binding site forward | 5’- GAGCGTCACTTCCGCCATCC -3’ |
| Binding site reverse | 5’- GAGGCCCCTCGGGAAATGTA -3’ |
